# Supplementary material for: Stachydrine, a Bioactive Equilibrist for Synephrine, Identified from Four Citrus Chinese Herbs
Source: Molecules. 2023 Apr 29;28(9):3813. doi: 10.3390/molecules28093813 (PMC10180305; doi:10.3390/molecules28093813)
Supplement: Supplementary file 1 [file molecules-28-03813-s001.zip › molecules-2331510-supplementary.pdf]

Supplementary Materials

Stachydrine, a bioactive equilibrist for synephrine, identified from four *Citrus* Chinese herbs

Yifei Sun [1,2], Xuexue Xia [1,2], Ganjun Yuan [1,2,*], Tongke Zhang [1], Beibei Deng [2], Xinyu Feng [2] and Qixuan Wang [2]

1 Biotechnological Engineering Center for Pharmaceutical Research and Development, Jiangxi Agricultural University, Nanchang 330045, China; sf857219493@163.com (Y.S.); xiaxuexue007@163.com (X.X.); ztk2628925331@sohu.com (T.Z.)
2 Laboratory of Natural Medicine and Microbiological Drug, College of Bioscience and Bioengineering, Jiangxi Agricultural University, Nanchang 330045, China; dengbeibei0412@163.com (B.D.); Fhoney18_5@163.com (X.F.); melo000120@126.com (Q.W.)
* Correspondence: gyuan@jxau.edu.cn; Tel.: +86-0791-83813459

Figure S1. Color development reactions of choline (**1**), synephrine (**2**) and γ-aminobutyric acid (**3**) on thin layer plates.

Figure S2. The linearity correlations between the amounts (*x*) and peak areas (*y*), of stachydrine (**a**), choline (**b**) and synephrine (**c**).

Figure S3**.** Detection of choline analogs in samples originated from *Citrus* genus plants on thin layer plates.

Figure S4**.** Detection of choline analogs in samples originated from other plants on thin layer plates.

Figure S5. ^{1}H (up), ^{13}C (middle) NMR and MS (down) spectra of compound **2**.

Table S1. The recovery of stachydrine (n = 9).

Table S2. The recovery of choline (n = 9).

Table S3. The recovery of synephrine (n = 9).

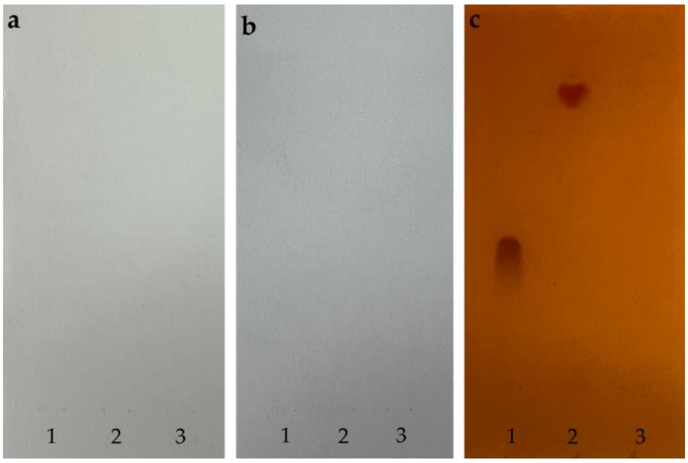

Figure S1**.** Color development reaction of choline (1), synephrine (2) and γ-aminobutyric acid (3) on thin layer plate. **a**: Dragendorff's reagent; **b**: Wagner reagent; **c**: improved Dragendorff's reagent.

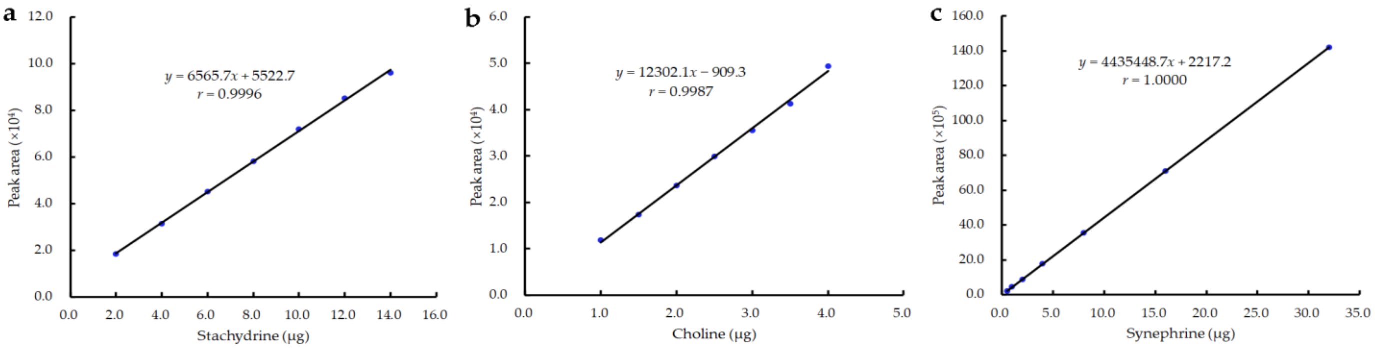

Figure S2. The linearity correlations between the amounts (x) and peak areas (y), of stachydrine (**a**), choline (**b**) and synephrine (**c**).

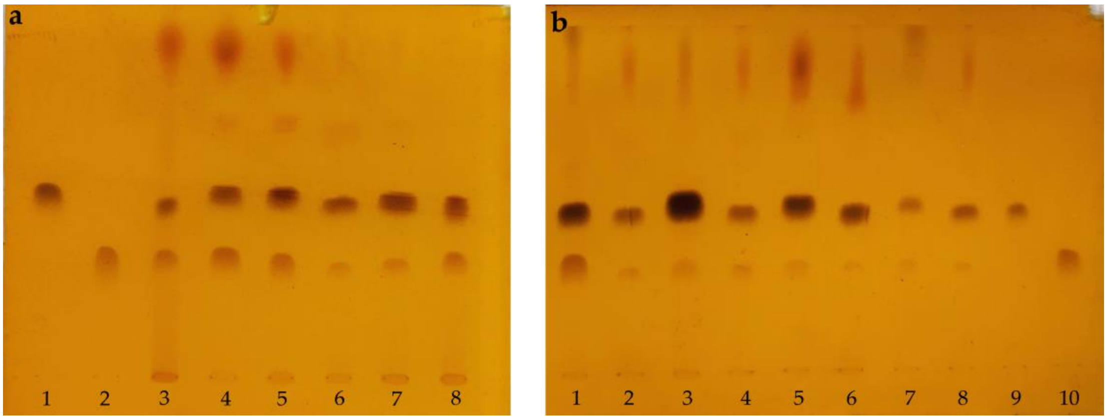

Figure S3. Detection of choline analogs in samples originated from *Citrus* genus plants on thin layer plates. a: 1, stachydrine; 2, choline; and samples from herbs 3, *Chenpi*; 4, *Qingpi*; 5, *Zhishi*; 6, *Zhiqiao*; 7, *Xiangyuan*; and 8, *Foshou*, respectively. b: samples from the leaves (1) and fruits (2) of *Citrus aurantium* L.; from the leaves (3) and fruits (4) of *Citrus junos* Siebold ex Tanaka; from the leaves (5) and fruits (6) of *Citrus reticulata* Blanco 'Zhangtouhong'; from the leaves (7) and fruits (8) of the cultivated variety of *Citrus aurantium* L 'Chouchen'; (9) stachydrine and (10) choline.

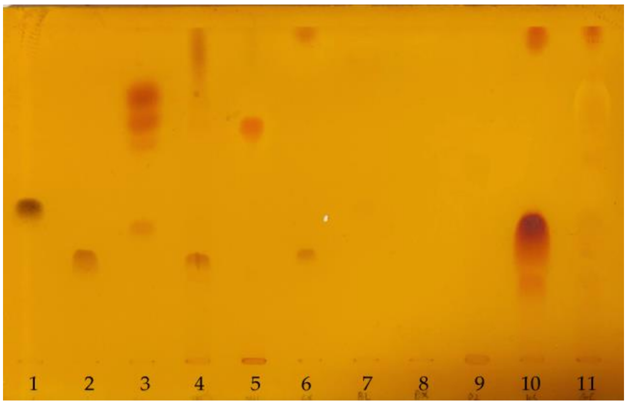

Figure S4. Detection of choline analogs in samples originated from other plants on thin layer plates. 1, stachydrine; 2, choline; and herbs 3, *Huanglian*; 4, *Juhua*; 5, *Mahuan*; 6, *Chuanxiong*; 7, *Dafupi*; 8, *Banxia*; 9, *Duzhong*; 10, *Kushen*; and 11, *Gancao*.

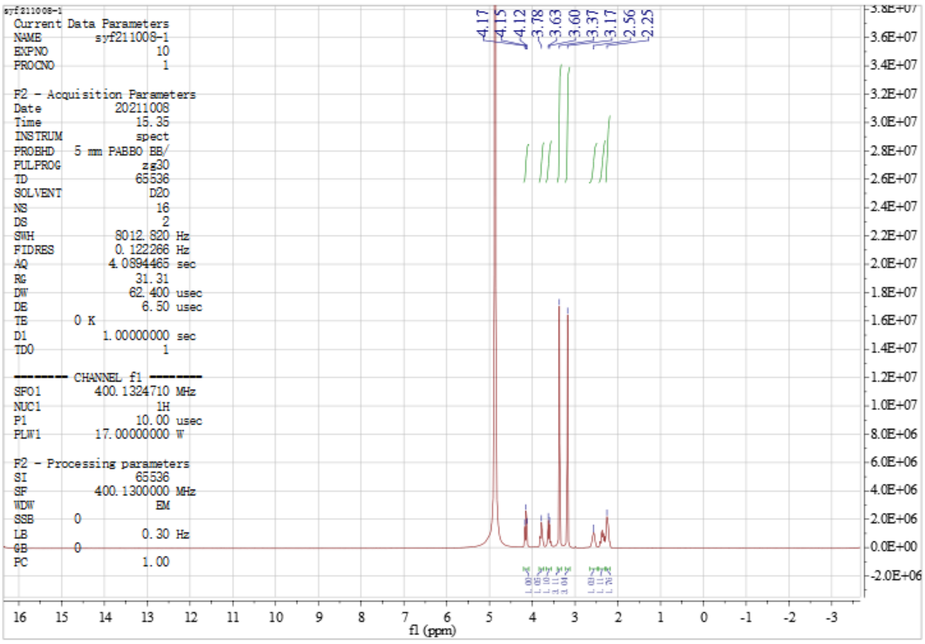

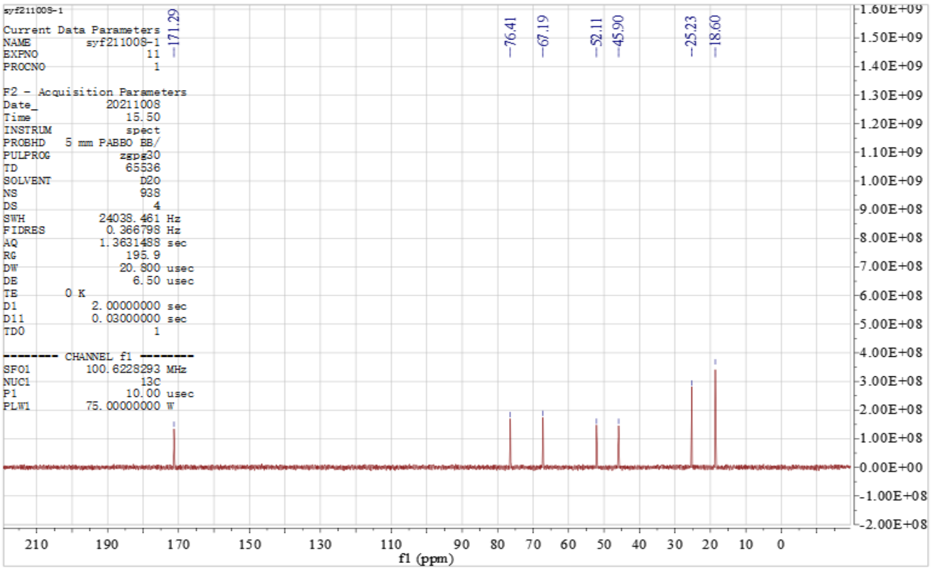

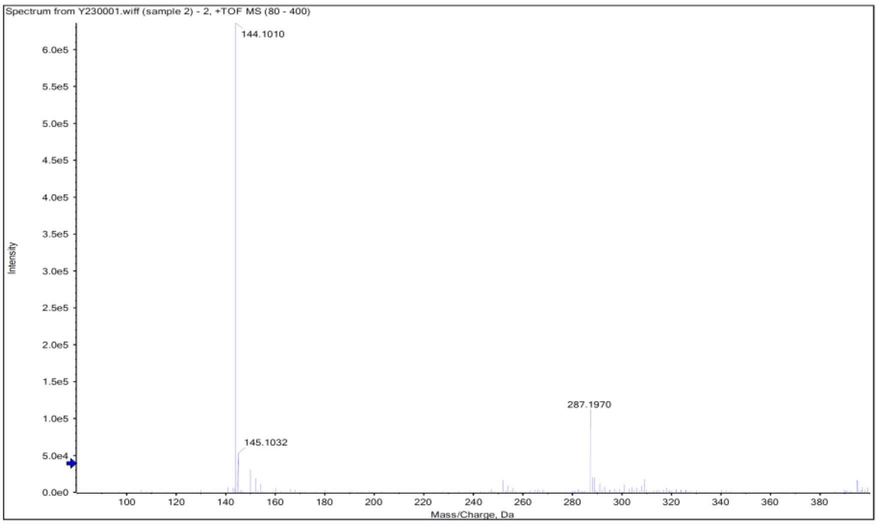

Figure S5. ^{1}H (up), ^{13}C (middle) NMR and MS (down) spectra of compound **2**.

Table S1. The recovery of stachydrine (n = 9).

Sample power (g)	Stachydrine in sample (mg)	Stachydrine added (mg)	Stachydrine tested (mg)	Recovery (%)	Average recovery (%)	RSD (%)
0.5003	1.54	6.50	8.06	101.30		
0.5004	1.50	6.50	7.96	97.33		
0.5001	1.48	6.50	8.03	103.38		
1.0001	2.90	5.00	7.95	101.72		
1.0003	3.10	5.00	8.09	99.68	100.28	3.08
1.0003	3.03	5.00	8.14	103.63		
1.4999	4.57	3.50	7.86	95.40		
1.5003	4.40	3.50	8.04	103.18		
1.5001	4.56	3.50	7.92	96.93		

Table S2. The recovery of choline (n = 9).

Sample power (g)	Choline in sample (mg)	Choline added (mg)	Choline tested (mg)	Recovery (%)	Average recovery (%)	RSD (%)
0.5002	0.41	2.60	3.02	102.44		
0.5003	0.42	2.60	3.01	97.62		
0.5001	0.44	2.60	3.03	97.73		
1.0004	0.84	2.20	3.05	101.19		
1.0005	0.86	2.20	3.09	103.49	101.04	2.31
1.0001	0.85	2.20	3.07	102.35		
1.5002	1.31	1.70	3.06	103.82		
1.5000	1.29	1.70	3.01	101.55		
1.4998	1.27	1.70	2.96	99.21		

Table S3. The recovery of synephrine (n = 9).

Sample power (g)	Synephrine in sample (mg)	Synephrine added (mg)	Synephrine tested (mg)	Recovery (%)	Average recovery (%)	RSD (%)
0.1000	1.10	0.80	1.95	104.54		
0.1003	1.11	0.80	1.94	102.70		
0.1001	1.08	0.80	1.90	101.85		
0.1001	1.12	1.00	2.07	95.53		
0.1000	1.08	1.00	2.05	97.22	99.89	3.19
0.1003	1.08	1.00	2.07	99.07		
0.1002	1.10	1.20	2.26	96.36		
0.1001	1.06	1.20	2.25	99.05		
0.1005	1.11	1.20	2.34	102.70		
